# Initial findings from a novel population-based child mortality surveillance approach: a descriptive study

**DOI:** 10.1016/S2214-109X(20)30205-9

**Published:** 2020-06-17

**Authors:** Allan W Taylor, Dianna M Blau, Quique Bassat, Dickens Onyango, Karen L Kotloff, Shams El Arifeen, Inacio Mandomando, Richard Chawana, Vicky L Baillie, Victor Akelo, Milagritos D Tapia, Navit T Salzberg, Adama Mamby Keita, Timothy Morris, Shailesh Nair, Nega Assefa, Anna C Seale, J Anthony G Scott, Reinhard Kaiser, Amara Jambai, Beth A Tippet Barr, Emily S Gurley, Jaume Ordi, Sherif R Zaki, Samba O Sow, Farzana Islam, Afruna Rahman, Scott F Dowell, Jeffrey P Koplan, Pratima L Raghunathan, Shabir A Madhi, Robert F Breiman, CHAMPS Consortium, Sozinho Acácio, Yasmin Adam, Sara Ajanovic, Muntasir Alam, Rebecca Alkis Ramirez, Henry Badji, Sanwarul Bari, J. Patrick Caneer, Atique Iqbal Chowdhury, Maureen H. Diaz, Karen D. Fairchild, Meerjady Sabrina Flora, Mischka Garel, Adriana Gibby, Nelesh P. Govender, Carol L. Greene, Martin John Hale, Juan Carlos Hurtado, J. Kristie Johnson, Mohammed Kamal, Tatiana Keita, Rima Koka, Diakaridia Koné, Sanjay G. Lala, Hennie Lombaard, Rita Mabunda, Roosecelis B. Martines, Ashka Mehta, Clara Menéndez, Sibone Mocumbi, Claudia Moya, Tacilta Nhampossa, Uma U. Onwuchekwa, Shahana Parveen, Karen L. Petersen, Rebecca Pass Phillipsborn, Mustafizur Rahman, Natalia Rakislova, Jana Ritter, Hossain M.S. Sazzad, Diakaridia Sidibe, Antonio Sitoe, Kasthuri Sivalogan, Jennifer M. Swanson, Peter J. Swart, Sharon M. Tennant, Cheick B. Traoré, Rosauro Varo Cobos, Pio Vitorino, Marta Valente, Sithembiso Velaphi, Jeannette Wadula, Jessica L. Waller, Amanda L. Wilkinson, Jonas M. Winchell

**Affiliations:** a Center for Global Health, Centers for Disease Control and Prevention, Atlanta, GA, USA; b National Center for Emerging and Zoonotic Infectious Diseases, Centers for Disease Control and Prevention, Atlanta, GA, USA; c ISGlobal, Hospital Clínic, University of Barcelona, Barcelona, Spain; d Centro de Investigação em Saúde de Manhiça (CISM), Maputo, Mozambique; e Catalan Institution for Research and Advanced Studies (ICREA), Barcelona, Spain; f Pediatrics Department, Pediatric Infectious Diseases Unit, Hospital Sant Joan de Déu, Barcelona, Spain; g Consorcio de Investigación Biomédica en Red de Epidemiología y Salud Pública (CIBERESP), Madrid, Spain; h Medical Research Council, Respiratory and Meningeal Pathogens Research Unit, School of Pathology and Department of Science and Technology/National Research Foundation: Vaccine Preventable Diseases Unit, Faculty of Health Sciences, University of the Witwatersrand, Johannesburg, South Africa; i Kisumu County Department of Health, Kisumu, Kenya; j Department of Pediatrics, Center for Vaccine Development and Global Health and Division of Infectious Disease and Tropical Pediatrics, University of Maryland School of Medicine, Baltimore, MD, USA; k International Centre for Diarrhoeal Disease Research, Dhaka, Bangladesh; l CDC Kenya, Kisumu, Kenya; m Emory Global Health Institute, Emory University, Atlanta, GA, USA; n Centre for Vaccine Development, Bamako, Mali; o Public Health Informatics Institute, Task Force for Global Health, Atlanta, GA, USA; p College of Health and Medical Sciences, Haramaya University, Harar, Ethiopia; q London School of Hygiene & Tropical Medicine, London, UK; r CDC Sierra Leone, Freetown, Sierra Leone; s Ministry of Health and Sanitation, Freetown, Sierra Leone; t Johns Hopkins Bloomberg School of Public Health, Johns Hopkins University, Baltimore, MD, USA; u Bill & Melinda Gates Foundation, Seattle, WA, USA

## Abstract

**Background:**

Sub-Saharan Africa and south Asia contributed 81% of 5·9 million under-5 deaths and 77% of 2·6 million stillbirths worldwide in 2015. Vital registration and verbal autopsy data are mainstays for the estimation of leading causes of death, but both are non-specific and focus on a single underlying cause. We aimed to provide granular data on the contributory causes of death in stillborn fetuses and in deceased neonates and children younger than 5 years, to inform child mortality prevention efforts.

**Methods:**

The Child Health and Mortality Prevention Surveillance (CHAMPS) Network was established at sites in seven countries (Baliakandi, Bangladesh; Harar and Kersa, Ethiopia; Siaya and Kisumu, Kenya; Bamako, Mali; Manhiça, Mozambique; Bombali, Sierra Leone; and Soweto, South Africa) to collect standardised, population-based, longitudinal data on under-5 mortality and stillbirths in sub-Saharan Africa and south Asia, to improve the accuracy of determining causes of death. Here, we analysed data obtained in the first 2 years after the implementation of CHAMPS at the first five operational sites, during which surveillance and post-mortem diagnostics, including minimally invasive tissue sampling (MITS), were used. Data were abstracted from all available clinical records of deceased children, and relevant maternal health records were also extracted for stillbirths and neonatal deaths, to incorporate reported pregnancy or delivery complications. Expert panels followed standardised procedures to characterise causal chains leading to death, including underlying, intermediate (comorbid or antecedent causes), and immediate causes of death for stillbirths, neonatal deaths, and child (age 1–59 months) deaths.

**Findings:**

Between Dec 10, 2016, and Dec 31, 2018, MITS procedures were implemented at five sites in Mozambique, South Africa, Kenya, Mali, and Bangladesh. We screened 2385 death notifications for inclusion eligibility, following which 1295 families were approached for consent; consent was provided for MITS by 963 (74%) of 1295 eligible cases approached. At least one cause of death was identified in 912 (98%) of 933 cases (180 stillbirths, 449 neonatal deaths, and 304 child deaths); two or more conditions were identified in the causal chain for 585 (63%) of 933 cases. The most common underlying causes of stillbirth were perinatal asphyxia or hypoxia (130 [72%] of 180 stillbirths) and congenital infection or sepsis (27 [15%]). The most common underlying causes of neonatal death were preterm birth complications (187 [42%] of 449 neonatal deaths), perinatal asphyxia or hypoxia (98 [22%]), and neonatal sepsis (50 [11%]). The most common underlying causes of child deaths were congenital birth defects (39 [13%] of 304 deaths), lower respiratory infection (37 [12%]), and HIV (35 [12%]). In 503 (54%) of 933 cases, at least one contributory pathogen was identified. Cytomegalovirus, *Escherichia coli*, group B Streptococcus, and other infections contributed to 30 (17%) of 180 stillbirths. Among neonatal deaths with underlying prematurity, 60% were precipitated by other infectious causes. Of the 275 child deaths with infectious causes, the most common contributory pathogens were *Klebsiella pneumoniae* (86 [31%]), *Streptococcus pneumoniae* (54 [20%]), HIV (40 [15%]), and cytomegalovirus (34 [12%]), and multiple infections were common. Lower respiratory tract infection contributed to 174 (57%) of 304 child deaths.

**Interpretation:**

Cause of death determination using MITS enabled detailed characterisation of contributing conditions. Global estimates of child mortality aetiologies, which are currently based on a single syndromic cause for each death, will be strengthened by findings from CHAMPS. This approach adds specificity and provides a more complete overview of the chain of events leading to death, highlighting multiple potential interventions to prevent under-5 mortality and stillbirths.

**Funding:**

Bill & Melinda Gates Foundation.

## Introduction

Sub-Saharan Africa and south Asia contributed 81% of 5·9 million under-5 deaths^1^ and 77% of 2·6 million stillbirths worldwide in 2015.^2^ Although mortality among children aged younger than 5 years has declined globally in the past two decades,^3^ considerable geographical differences exist. Child mortality remains disproportionately high in sub-Saharan Africa and south Asia.^1^ Understanding the aetiologies of child mortality is hindered by a high proportion of child deaths at the community level, inadequate access to medical services, scarcity of pre-mortem and post-mortem diagnostic tools, coexisting illnesses (which are difficult to distinguish using available tools), absence of functional vital registration systems, and insufficient validity and precision of common methods for cause of death determination.^4^, ^5^, ^6^, ^7^, ^8^, ^9^ The paucity of credible, reliable mortality data limits national capabilities to optimally prioritise scarce resources for reducing mortality.^10^

**Research in context**

**Evidence before this study**

In low-income and middle-income countries (LMICs), data used for estimating cause of death distributions for populations are primarily based on verbal autopsy interviews and, occasionally, death certificates using hospital-based clinical data, both of which have limited accuracy. There is poor concordance between findings from verbal autopsy or death certificates and the diagnoses obtained from complete diagnostic autopsy, which is considered the gold standard for cause of death investigation. Complete diagnostic autopsies, however, pose challenges in many settings, including limited cultural and religious acceptability, because they might be considered disfiguring and time consuming and can substantially delay burial. Furthermore, in many LMICs, qualified personnel and other resources required for the procedure are scarce. Considering that the majority of global child mortality occurs in LMICs, an acceptable and feasible approach that would inform reliably on causes and the chain of events leading to death in a selection of high mortality areas would generate more robust and credible data, thus providing the basis for more accurate and specific estimates of childhood mortality.

**Added value of this study**

The Child Health and Mortality Prevention and Surveillance Network includes sites in sub-Saharan Africa and south Asia, with the joint goal of generating and accumulating more precise, detailed, and robust data on causes of child mortality in locations across these regions with high child mortality. Deaths were investigated using a variety of methodologies, including minimally invasive tissue sampling (MITS), a post-mortem approach using biopsy needles for sampling key organs and body fluids, which has been validated as an acceptable proxy method to complete diagnostic autopsy for cause of death ascertainment. Molecular testing, classical microbiology, histopathology, and immunohistochemistry were used to assess samples obtained from MITS. Additionally, we used verbal autopsy and collected antemortem clinical data. In-country panels of experts analysed each death using all available data and determined the most plausible sequence and causes of death. In this study, we demonstrate the contribution of these methods for detailing cause of death data at a level of granularity previously unavailable in LMICs and show the high acceptance rate for the MITS procedure in a range of LMIC settings. We describe the first insights regarding the advantages of this approach for under-5 mortality surveillance, which could highlight potential pathways to prevent child mortality.

**Implications of all the available evidence**

The mortality surveillance approach described in this study has the potential to increase the precision and credibility of cause of death estimates. Rather than focusing on a singular underlying, often non-specific, cause of death, which might not fully inform interventions to reduce mortality, we have presented detailed descriptions of the underlying and immediate causes of death, which together with any antecedent conditions, comprise the chain of events leading to death. This information provides an improved understanding of conditions associated with death in regions with high child mortality. MITS with associated laboratory analysis facilitates the identification of specific microorganisms and thus provides a clearer understanding of the contribution of pathogens to the histopathological changes observed in each case and to overall child mortality. MITS data might also provide a foundation for the calibration and refinement of the interpretation of data from existing tools (especially verbal autopsy and death certificates) for future use in global burden of disease estimates and to better inform public health policy.

Minimally invasive tissue sampling (MITS), also known as minimally invasive autopsy, was developed to improve the accurate ascertainment of causes of death in resource-constrained settings.^11^ Compared with complete diagnostic autopsy, MITS is less physically disruptive, faster and potentially more socioculturally acceptable,^12^ and less resource-intensive.^13^ MITS has been validated against complete diagnostic autopsies in perinatal and paediatric deaths with International Classification of Diseases, tenth revision (ICD-10) diagnostic categories coinciding in 68–89% of cases (complete diagnostic autopsies *vs* MITS diagnoses).^14^, ^15^

The Child Health and Mortality Prevention Surveillance (CHAMPS) Network was established to collect standardised, population-based, longitudinal data within a network of sites in areas with high child mortality, with the overarching objectives of understanding and tracking preventable causes of childhood death and stillbirths globally.^16^ In this study, we analysed data obtained in the first 2 years after the implementation of CHAMPS to characterise the types of results that will contribute to burden of disease estimates, future policy making, advocacy, and research agendas, as data accumulate over time. We aimed to provide data from the first 2 years of CHAMPS activities on all under-5 deaths and stillbirths, using MITS to obtain detailed, high quality and pathogen-specific cause of death data.

## Methods

### Mortality and demographic surveillance

The CHAMPS Network included sites in seven countries: Baliakandi, Bangladesh; Harar and Kersa, Ethiopia; Siaya and Kisumu, Kenya; Bamako, Mali; Manhiça, Mozambique; Bombali, Sierra Leone; and Soweto, South Africa. Site characteristics and selection criteria have been described previously.^17^ As of Dec 31, 2018, five sites had implemented minimally invasive tissue sampling, and data from these sites are included in this report. Some sites used pre-existing Health and Demographic Surveillance System (HDSS) catchment areas.^18^, ^19^, ^20^, ^21^ New surveillance areas were developed for CHAMPS within informal settlements in Kisumu, Baliakandi, and Soweto. The total population size for catchment areas within each country (with the exception of South Africa) ranged between 170 000 and 227 219 individuals.^21^ The Soweto site, in the absence of an established HDSS, used the entire township and surrounding informal settlement areas (population of approximately 1·3 million people) as the catchment area during the majority of the reported period while the HDSS was being established. Sites built relationships with communities^22^ and health facilities within catchment areas, posting CHAMPS staff at health facilities and developing networks of community reporters to receive notifications of under-5 deaths and stillbirths within the first 24 h.^17^ At most sites, death notifications were initially mostly received from primary referral health facilities, subse-quently expanding to notifications of deaths occurring outside health facilities.

After receiving notifications of deaths or stillbirths, CHAMPS staff approached families rapidly for eligibility screening. Eligible cases were aged younger than 60 months, and they or their parents resided within the study catchment area. MITS eligibility required that CHAMPS staff were notified within 36 h of death (or ≤72 h if post-mortem refrigeration used) and that the body of the stillborn fetus or deceased child was available for analysis. Families provided consent for the MITS procedure, verbal autopsy, and clinical data abstraction, and we present data on these MITS-eligible fetuses, neonates, or children in this report. Stillborn fetuses and deceased neonates and infants who were not eligible for the MITS procedure (eg, CHAMPS received a notification more than 36 h after their death or consent for MITS was not given) were asked to consent for only verbal autopsy interview and clinical data abstraction, and will be reported elsewhere.^17^

Parents or guardians of stillborn fetuses or deceased children provided written informed consent before collection of data, specimens, or information on the mothers. Ethics committees overseeing investigators at each site and at Emory University (Atlanta, GA, USA) approved overall and site-specific protocols, as appropriate. The Centers for Disease Control and Prevention (CDC; Atlanta, GA, USA) relied on the Emory University committee to review the overall protocol and on appropriate ethical review committees at the sites where CDC staff were directly engaged. Protocols have been published previously.

### Specimen and data collection

Trained CHAMPS staff took photographs to identify dysmorphic features and took anthropometric measurements before specimen collection. In sterile conditions, tissue specimens were collected using biopsy needles from the lungs, heart, brain, liver, and bone marrow.^23^ They also collected peripheral blood, cerebrospinal fluid, stool, and nasopharyngeal secretions. Site laboratories tested post-mortem blood samples for HIV DNA or RNA by PCR, malaria thick and thin smears, and rapid diagnostic tests; blood and cerebrospinal fluid were cultured. Five custom TaqMan Array Cards (ThermoFisher Scientific, Waltham, MA, USA) with specific molecular assays were used to detect 116 pathogen targets from lung tissue, blood, cerebrospinal fluid, rectal brush (collecting stool), and nasopharyngeal swabs.^23^, ^24^ Pathology laboratories at each site and at the CDC applied histopathology to all tissues, including routine stains. Special stains, such as tissue Gram stain, targeting microorganisms and immunohistochemistry were done when relevant, at pathology laboratories at the CDC.^25^ Data were abstracted from all available clinical records of deceased children and relevant maternal health records were abstracted for stillbirths, neonatal deaths, and others with reported pregnancy or delivery complications. Trained interviewers used appropriate-language translations of the 2016 WHO verbal autopsy instrument^17^, ^26^ to interview caregivers of enrolled deceased children within 4 weeks of death, when feasible.

### Cause of death determination

All data available for each case were reviewed by Determination of Cause of Death (DeCoDe) panels convened at each site, which consisted of paediatricians, obstetricians, other health-care providers, epidemiologists, pathologists, and microbiologists.^27^ The panels reviewed available case data and determined the chain of events leading to death using WHO ICD-10 and WHO application of ICD-10 to deaths during the perinatal period (ICD-PM) guidelines.^28^, ^29^ For cases in which more than one condition led to death, the process included documenting the entire causal chain leading to death, as in a standard death certificate, including underlying cause, intermediate (referred to on the WHO standard death certificate as comorbid or antecedent causes), and immediate cause. To be included in the causal chain, the panels considered whether appropriate management or prevention of that condition could have prevented the death. Additionally, the panels identified any other contributing causes of death and the main maternal factors contributing to perinatal deaths. The immediate cause of death was the most proximal cause leading to death, and antecedent or comorbid causes were others contributing directly to the chain of events leading to death. The underlying cause was the disease or injury that initiated the chain of events that led to the death. Each case could have one immediate cause and multiple comorbid or antecedent causes identified, which could be in the same general category (eg, lower respiratory tract infection could be listed twice in the same causal chain if, for example, a viral pneumonia led to a bacterial superinfection). Additionally, the panels considered all available data for evidence of signs of life (such as postnatal respiration, heartbeat, or movement); the presence of any resulted in a case being categorised as a neonatal death rather than a stillbirth. The DeCoDe process was standardised across the network using diagnosis standards for defining and coding common childhood causes of death and organisation of specific and homogeneous training at all sites.^27^ Causes were grouped into standardised categories for analysis (appendix p 2). All analyses were conducted using R (version 3.6.3).

### Role of the funding source

The funder of the study provided input on study design and site selection and suggested edits on the final versions of the manuscript, but had no role in data collection, data analysis, or data interpretation. The corresponding author had full access to all the data in the study and had final responsibility for the decision to submit for publication.

## Results

Between Dec 10, 2016, and Dec 31, 2018, MITS procedures were implemented at five sites (in Bangladesh, Kenya, Mali, Mozambique, and South Africa); 2385 case notifications of stillborn or deceased children were screened and determined to be eligible for data collection (figure). Surveillance staff were notified within 36 h of death for 1749 (73%) of 2385 eligible stillborn fetuses or deceased children, of which 1605 (92%) notifications were received within 24 h (appendix p 15). Eligible deaths that occurred in health facilities were more likely to be notified within 24 h than deaths occurring outside of health facilities (1360 [74%] of 1755 deaths in health facilities *vs* 233 [43%] of 536 deaths outside of health facilities). Median time from death to notification was 13·0 h (IQR 2·0–47·5).

**Figure:**
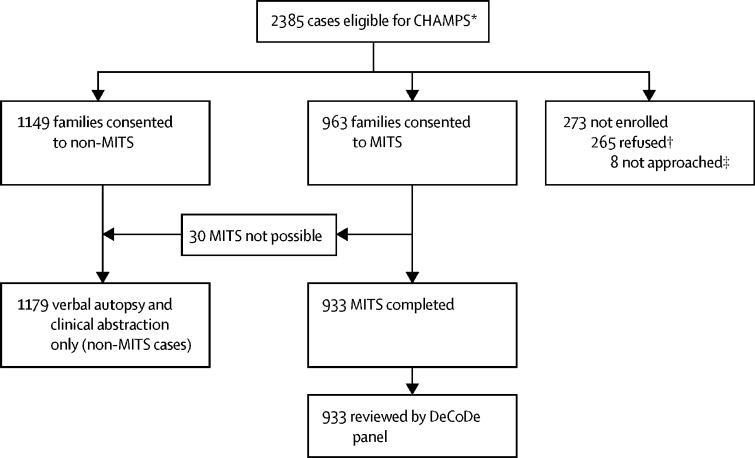
Case selection process for CHAMPS CHAMPS=Child Health and Mortality Prevention Surveillance. MITS=minimally invasive tissue sampling. DeCoDe=Determination of Cause of Death. *Does not include known cases outside of catchment areas, not available for eligibility screening, or considered lost to follow-up. †Cases that were approached for MITS, non-MITS, or MITS followed by non-MITS consent and refused. ‡Cases that were considered MITS eligible, but were not approached for either MITS or non-MITS consent.

Of 2385 cases eligible for inclusion in CHAMPS, 1430 were eligible for MITS. Of the 1430 eligible cases, the families of 1295 (91%) cases were approached for MITS consent, of whom 963 (74%) families consented to MITS (189 [80%] among 236 stillbirths, 468 [71%] among 662 neonates, and 306 [77%] among 397 children aged 1–59 months; figure). Of all 963 cases for whom we received parental consent for inclusion, MITS was completed in 933 cases (180 stillbirths, 449 neonatal deaths, 304 children's deaths). MITS was not done in 30 cases after we received familial consent because the body of the fetus or child was no longer available after consent (n=17); pathologists were not available for the procedure (n=5); the specimen collection kit was not available (n=3); consent was withdrawn (n=3); or the body of the child or fetus was not in sufficient condition to test (n=2). MITS was completed within 24 h of death for 616 (66%) of 933 cases (appendix p 15); in two cases, MITS was done more than 72 h after death. Surveillance was initiated on different dates at each of the five sites during the study period; all sites started by primarily including deaths that had occurred at health facilities. Overall, 832 (89%) of 933 deaths occurred at health facilities. 464 (50%) of 933 cases were from the Soweto site (which had a disproportionately larger catchment area in 2017 and began collecting MITS earlier than three other sites), in which the main medical facility is a large hospital with a large neonatal intensive care unit (table 1). DeCoDe panel assessments of the 933 cases formed the basis for this descriptive analysis. Overall, among the 933 cases, at least one cause of death was identified in 912 (98%) of cases, and in 585 (63%) of cases, we identified two or more conditions in the causal chain (appendix p 17). In 503 (54%) of 933 of cases, at least one contributory pathogen was identified.

**Table 1:** Characteristics of cases with complete cause of death determination, by CHAMPS Network site

	**South Africa (n=464)**	**Mozambique (n=146)**	**Kenya (n=182)**	**Mali (n=95)**	**Bangladesh (n=46)**	**Total (n=933)**
**Age group**						
Stillbirth	41 (9%)	48 (33%)	39 (21%)	31 (33%)	21 (46%)	180 (19%)
Death in the first 24 h	81 (17%)	35 (24%)	20 (11%)	15 (16%)	11 (24%)	162 (17%)
Early neonate (1 to 6 days)	122 (26%)	16 (11%)	20 (11%)	17 (18%)	10 (22%)	185 (20%)
Late neonate (7 to 27 days)	74 (16%)	5 (3%)	12 (7%)	8 (8%)	3 (7%)	102 (11%)
Infant (28 days to <12 months)	98 (21%)	15 (10%)	48 (26%)	13 (14%)	0	174 (19%)
Child (12 months to less than 60 months)	48 (10%)	27 (18%)	43 (24%)	11 (12%)	1 (2%)	130 (14%)
**Religion of primary caregiver**						
Muslim	1 (<1%)	1 (1%)	0	95 (100%)	38 (83%)	135 (14%)
Christian	303 (65%)	82 (56%)	172 (95%)	0	0	557 (60%)
African traditional religion	3 (1%)	32 (22%)	10 (5%)	0	0	45 (5%)
Other or no religion	157 (34%)	31 (21%)	0	0	8 (17%)	196 (21%)
**Sex of deceased fetus, neonate, or child**						
Female	186 (40%)	62 (42%)	91 (50%)	50 (53%)	18 (39%)	407 (44%)
Male	277 (60%)	84 (58%)	90 (49%)	45 (47%)	28 (61%)	524 (56%)
Unknown	1 (<1%)	0	1 (1%)	0	0	2 (<1%)
**Time from death to notification, h**						
≤24	360 (78%)	146 (100%)	166 (91%)	95 (100%)	46 (100%)	813 (87%)
>24	104 (22%)	0	16 (9%)	0	0	120 (13%)
**Time from death to MITS procedure, h**						
≤24	238 (51%)	133 (91%)	104 (57%)	95 (100%)	46 (100%)	616 (66%)
>24	225 (48%)	13 (9%)	78 (43%)	0	0	316 (34%)
Unknown	1 (<1%)	0	0	0	0	1 (0%)
**Location and timing of death**						
Facility (≤24 h after admission)	127 (27%)	53 (36%)	49 (27%)	23 (24%)	16 (35%)	268 (29%)
Facility (>24 h after admission)	318 (69%)	84 (58%)	89 (49%)	51 (54%)	30 (65%)	572 (61%)
Community	19 (4%)	9 (6%)	44 (24%)	21 (22%)	0 (0%)	93 (10%)

Data are n (%). CHAMPS=Child Health and Mortality Prevention Surveillance. MITS=minimally invasive tissue sampling.

A specific cause leading to fetal death was identified in 172 (96%) of 180 stillbirths (appendix p 17). Of 180 stillbirths, perinatal asphyxia or hypoxia was the most frequently identified underlying cause of death (130 [72%] cases), with congenital infection or sepsis the second most common underlying cause (27 [15%] cases; table 2). A pathogen was determined to have contributed to the causal chain leading to stillbirth in 30 (17%) of 180 cases (data not shown). The most common contributory pathogens were cytomegalovirus (n=7), *Escherichia coli* or *Shigella* (n=6), *Streptococcus agalactiae* (n=6), and *Enterococcus faecalis* (n=5; table 3). An illustrative stillbirth case is presented in the appendix (p 19). Among 172 stillbirths for which a cause was determined, a maternal or obstetric factor was identified in 154 (90%) of cases. Using ICD-PM classifications,^28^ the most common maternal factors were complications of placenta, cord, and membranes (66 [43%] of 154 cases) and maternal medical and surgical conditions (54 [35%] of 154 cases), predominantly associated with hypertensive disorders, such as pre-eclampsia and eclampsia (table 4; appendix p 18).

**Table 2:** All identified cause of death categories, by age group and position in causal chain

	**Immediate cause or comorbid cause**	**Underlying cause**	**Immediate, comorbid, or underlying cause**
**Stillbirths (n=180)**			
Perinatal asphyxia or hypoxia	5 (3%)	130 (72%)	135 (75%)
Congenital infection or sepsis	7 (4%)	27 (15%)	30 (17%)
Congenital birth defects	2 (1%)	7 (4%)	8 (4%)
Other neonatal disorders	6 (3%)	2 (1%)	8 (4%)
Neonatal preterm birth complications	4 (2%)	1 (1%)	5 (3%)
Umbilical cord complications	1 (1%)	1 (1%)	2 (1%)
Placental complications	0	2 (1%)	2 (1%)
Syphilis	0	2 (1%)	2 (1%)
Birth trauma	1 (1%)	0	1 (1%)
Malpresentation before labour	1 (1%)	0	1 (1%)
**Neonatal deaths (n=449)**			
Preterm birth complications	182 (41%)	187 (42%)	227 (51%)
Sepsis	134 (30%)	50 (11%)	181 (40%)
Perinatal asphyxia or hypoxia	29 (6%)	98 (22%)	127 (28%)
Lower respiratory tract infections	86 (19%)	4 (1%)	90 (20%)
Meningitis or encephalitis	61 (14%)	4 (1%)	65 (14%)
Other neonatal disorders	52 (12%)	9 (2%)	60 (13%)
Congenital birth defects	13 (3%)	39 (9%)	47 (10%)
Congenital infection	27 (6%)	20 (4%)	46 (10%)
Neonatal encephalopathy	34 (8%)	7 (2%)	41 (9%)
Neonatal aspiration syndromes	10 (2%)	5 (1%)	15 (3%)
Other respiratory disease	9 (2%)	0	9 (2%)
Kidney disease	8 (2%)	0	8 (2%)
Other infections	7 (2%)	1 (<1%)	8 (2%)
Other	4 (1%)	1 (<1%)	5 (1%)
Liver disease	3 (1%)	0	3 (1%)
Chorioamnionitis and membrane complications	0	3 (1%)	3 (1%)
Birth trauma	2 (<1%)	0	2 (<1%)
Paralytic ileus and intestinal obstruction	2 (<1%)	0	2 (<1%)
Other disorders of fluid, electrolyte, and acid-base balance	1 (<1%)	1 (<1%)	2 (<1%)
Placental complications	1 (<1%)	1 (<1%)	2 (<1%)
Umbilical cord complications	1 (<1%)	1 (<1%)	2 (<1%)
HIV	0	2 (<1%)	2 (<1%)
Syphilis	0	2 (<1%)	2 (<1%)
Liver disease	1 (<1%)	0	1 (<1%)
Maternal HIV	1 (<1%)	0	1 (<1%)
Other endocrine, metabolic, blood, and immune disorders	1 (<1%)	0	1 (<1%)
Caesarean delivery	0	1 (<1%)	1 (<1%)
Diarrheal diseases	0	1 (<1%)	1 (<1%)
Injury	0	1 (<1%)	1 (<1%)
Maternal hypertension	0	1 (<1%)	1 (<1%)
Maternal infection	0	1 (<1%)	1 (<1%)
Measles	0	1 (<1%)	1 (<1%)
Obstructed labour and fetal malpresentation	0	1 (<1%)	1 (<1%)
Child deaths*(n=304)			
Lower respiratory tract infections	143 (47%)	37 (12%)	174 (57%)
Sepsis	119 (39%)	6 (2%)	124 (41%)
Malnutrition	18 (6%)	31 (10%)	49 (16%)
Congenital birth defects	13 (4%)	39 (13%)	45 (15%)
Diarrhoeal diseases	19 (6%)	24 (8%)	43 (14%)
HIV	0	35 (12%)	35 (12%)
Preterm birth complications	15 (5%)	29 (10%)	32 (11%)
Malaria	7 (2%)	24 (8%)	31 (10%)
Meningitis or encephalitis	28 (9%)	2 (1%)	30 (10%)
Other infections	21 (7%)	6 (2%)	27 (9%)
Injury	2 (1%)	15 (5%)	17 (6%)
Other respiratory disease	12 (4%)	5 (2%)	16 (5%)
Other	8 (3%)	2 (1%)	10 (3%)
Other neurological disorders	7 (2%)	4 (1%)	10 (3%)
Liver disease	5 (2%)	3 (1%)	8 (3%)
Other endocrine, metabolic, blood, and immune disorders	6 (2%)	2 (1%)	7 (2%)
Congenital infection	2 (1%)	4 (1%)	6 (2%)
Anaemias	5 (2%)	0	5 (2%)
Poisoning	4 (1%)	1 (<1%)	5 (2%)
Heart diseases	3 (1%)	2 (1%)	5 (2%)
Tuberculosis	3 (1%)	2 (1%)	5 (2%)
Kidney disease	4 (1%)	0	4 (1%)
Other neonatal disorders	4 (1%)	0	4 (1%)
Paralytic ileus and intestinal obstruction	3 (1%)	2 (1%)	4 (1%)
Other skin and subcutaneous diseases	2 (1%)	2 (1%)	4 (1%)
Sickle cell disorders	1 (<1%)	3 (1%)	4 (1%)
Other disorders of fluid, electrolyte, and acid-base balance	3 (1%)	0	3 (1%)
Other immunodeficiencies	2 (1%)	1 (<1%)	3 (1%)
Epilepsy	1 (<1%)	2 (1%)	3 (1%)
Cancer	0	3 (1%)	3 (1%)
Maternal HIV	0	3 (1%)	3 (1%)
Neonatal encephalopathy	1 (<1%)	1 (<1%)	2 (1%)
Other injury	1 (<1%)	1 (<1%)	2 (1%)
Upper respiratory tract infections	1 (<1%)	1 (<1%)	2 (1%)
Measles	0	2 (1%)	2 (1%)
Liver disease	1 (<1%)	0	1 (<1%)
Other gastrointestinal disease	1 (<1%)	0	1 (<1%)
Motor neuron disease	0	1 (<1%)	1 (<1%)
Perinatal asphyxia or hypoxia	0	1 (<1%)	1 (<1%)
Road injuries	0	1 (<1%)	1 (<1%)
Sudden infant death syndrome	0	1 (<1%)	1 (<1%)

Data are n (%). Specific ICD-10 codes comprising each cause of death category are shown in the appendix (p 2). Cases with undetermined and unclassified causes were excluded from this table (eight stillbirths, seven neonatal deaths, and six child deaths). Counts in the immediate, comorbid, or underlying cause column do not always equal the sum of counts in the immediate or comorbid cause columns, since an individual case could have one or more diagnoses in a given category in both immediate or comorbid cause columns, but was counted as a single case in the immediate, comorbid, or underlying cause column.

* Age 1–59 months.

**Table 3:** Number of times pathogens were identified in causal chain leading to death, by condition and age group

	**Congenital infection (n)**	**Other condition (n)**	**Sepsis (n)**	**Lower respiratory tract infections (n)**	**Other infections (n)**	**Malaria (n)**	**Total instances of pathogen (n)**	**Unique cases with pathogen (n)**	**Cases in age group (%)**
**Stillbirths (n=180)**									
Cytomegalovirus	6	0	1	0	0	0	7	7	4%
*E coli* or *Shigell*a	4	0	6	0	0	0	10	6	3%
*S agalactiae*	2	1	4	0	0	0	7	6	3%
*E faecali*s	2	0	4	0	0	0	6	5	3%
Streptococcus	3	0	2	0	0	0	5	5	3%
*S pneumoniae*	1	0	2	0	0	0	3	2	1%
*Treponema pallidum*	2	0	0	0	0	0	2	2	1%
Coagulase-negative *staphylococcus*	0	0	1	0	0	0	1	1	1%
*Enterobacter*	0	0	1	0	0	0	1	1	1%
*Enterobacter cloacae*	1	0	v	0	0	0	1	1	1%
*H influenzae* type B	0	0	1	0	0	0	1	1	1%
*Klebsiella oxytoca*	1	0	1	0	0	0	2	1	1%
*K pneumoniae*	0	0	1	0	0	0	1	1	1%
*P aeruginos*a	1	0	0	0	0	0	1	1	1%
*Sneathia amnionii*	1	0	0	0	0	0	1	1	1%
**Neonatal deaths (n=449)**									
*A baumanni*i	0	27	77	50	1	0	155	97	22%
*K pneumoniae*	0	21	61	35	0	0	117	74	16%
*E coli* or *Shigella*	0	12	20	7	0	0	39	27	6%
S agalactiae	0	10	19	3	0	0	32	24	5%
*S aureu*s	0	2	10	7	0	0	19	16	4%
Streptococcus	0	5	2	6	0	0	13	12	3%
*E faecalis*	0	2	6	1	0	0	9	9	2%
*Candida albicans*	0	4	4	0	2	0	10	7	2%
*S pneumoniae*	0	2	3	3	0	0	8	7	2%
*Candida glabrata*	0	3	4	0	1	0	8	6	1%
Cytomegalovirus	0	6	0	0	1	0	7	6	1%
*Listeria monocytogenes*	0	7	0	0	0	0	7	6	1%
*Enterococcus faecium*	0	0	5	0	0	0	5	5	1%
*Candida parapsilosis*	0	3	1	0	0	0	4	4	1%
Candida	0	2	1	0	0	0	3	3	1%
Child deaths*(n=304)									
*K pneumoniae*	0	11	55	54	0	0	120	86	28%
*S pneumoniae*	0	7	22	46	0	0	75	54	18%
HIV	0	38	0	3	0	0	41	40	13%
Cytomegalovirus	4	12	4	24	0	0	44	34	11%
*Plasmodium falciparum*	0	0	0	0	0	27	27	30	10%
*A baumannii*	0	4	24	10	0	0	38	29	10%
*S aureus*	0	2	12	22	0	0	36	28	9%
*H influenzae*	0	1	5	19	0	0	25	20	7%
*E coli*	0	9	9	4	0	0	22	19	6%
Respiratory syncytial virus	0	1	0	17	0	0	18	18	6%
Adenovirus	0	5	2	11	0	0	18	16	5%
*Pneumocystis jirovecii*	0	1	1	17	0	0	19	16	5%
*P aeruginosa*	0	3	9	9	0	0	21	15	5%
Streptococcus	0	1	2	9	0	0	12	10	3%
Parainfluenza virus type 3	0	1	0	9	0	0	10	9	3%

The 15 most frequently identified pathogens for each age group are presented. The total number of conditions in the columns might exceed the total number of cases for each age group because cases could have more than one condition contributing to death. Cases with no pathogens identified in the causal chain were excluded from this table. *E coli*=*Escherichia coli. S agalactiae=Streptococcus agalactiae. E faecalis=Enterococcus faecalis. S pneumoniae=Streptococcus pneumoniae. H influenzae*=*Haemophilus influenzae. K pneumoniae=Klebsiella pneumoniae. P aeruginosa=Pseudomonas aeruginosa. A baumannii=Acinetobacter baumannii. S aureus=Staphylococcus aureus.*

* Age 1–59 months.

**Table 4:** Stillbirth cause by maternal factor

	**Maternal factor 1: complications of placenta, cord, and membranes**	**Maternal factor 2: maternal complications of pregnancy**	**Maternal factor 3: other complications of labour and delivery**	**Maternal factor 4: maternal medical and surgical conditions**	**Total**
Congenital malformations, deformations and chromosomal abnormalities	1 (1%)	0	1 (1%)	1 (1%)	3 (2%)
Birth trauma	0	0	0	0	0
Perinatal asphyxia or hypoxia	50 (32%)	10 (6%)	20 (13%)	44 (29%)	124 (81%)
Infection	15 (10%)	1 (1%)	0	9 (6%)	25 (16%)
Other specified antepartum or intrapartum disorder	0	0	1 (1%)	0	1 (1%)
Disorders related to fetal growth	0	1 (0·6%)	0	0	1 (1%)
Total	66 (43%)	12 (8%)	22 (14%)	54 (35%)	154 (100%)

Data are n (%). ICD-PM maternal condition groups listed in the appendix (p 18).

At least one cause of death was identified for 442 (98%) of 449 neonatal deaths, with more than one condition determined to be in the causal chain leading to death in 330 (73%) of 449 neonatal deaths (appendix p 17). Of the 449 neonatal deaths, the most common underlying cause categories were preterm birth complications (187 [42%]), perinatal asphyxia or hypoxia (98 [22%]), neonatal sepsis (50 [11%]), congenital birth defects (39 [9%]), and congenital infection (20 [4%]; table 2). The most frequent comorbid or antecedent causes and immediate causes included preterm birth complications (182 [41%]), neonatal sepsis (126 [28%]), lower respiratory tract infections (86 [19%]), and meningitis or encephalitis (61 [14%]; table 2).

Overall, 240 (53%) of 449 neonatal deaths involved at least one infectious condition in the causal chain: 224 (93%) of 240 deaths involved at least one contributory pathogen, and 151 (34%) of 449 deaths involved more than one pathogen (data not shown). Of the 240 neonatal deaths with at least one infectious condition in the causal chain, the most common contributory pathogens were *Acinetobacter baumannii* (97 [40%] of cases with an infectious condition), *Klebsiella pneumoniae* (74 [31%]), and *E coli* (27 [11%]; table 3). Among 88 neonates whose death was attributed to an infectious cause and who were admitted to hospital less than 48 h before death, the most common pathogens identified were *K pneumoniae* (n=19), *E coli* (n=17), and *S agalactiae* (n=14; data not shown). The most common conditions associated with pathogens were lower respiratory tract infections and sepsis (table 3). Of the 227 neonatal deaths involving preterm birth complications, 141 (62%) neonates had an identified infectious condition that contributed to their death (data not shown). A neonatal death that demonstrates the identification of the causative organism for a death due to pneumonia and sepsis in a premature neonate is presented in the appendix (p 19).

Of 304 child deaths, at least one cause of death was identified in 298 (98%) cases, and more than one cause of death was identified in 230 (76%) cases (appendix p 17). The most common underlying causes of death were congenital birth defects (39 [13%] of 304 cases), lower respiratory tract infection (37 [12%]), HIV (35 [12%]), malnutrition (31 [10%]), and preterm birth complications (29 [10%]; table 2). The most common causes of death across all causes (underlying cause, comorbid or antecedent causes, and immediate cause) were lower respiratory tract infections (174 [57%] of 304 cases) and sepsis (123 [40%]; table 2). Lower respiratory tract infections contributed to 59% of deaths resulting from malnutrition and to 62% of those resulting from congenital abnormalities (data not shown).

Overall, 275 (90%) of 304 child deaths had at least one infectious condition listed in the causal chain, among which at least one contributory pathogen was identified in 249 (91%) of 275 cases, and 196 (64%) of 304 cases involved more than one contributory pathogen. The most common contributory pathogens were *K pneumoniae* (86 [31%] of 275 deaths with infectious causes), *S pneumoniae* (54 [20%]), HIV (40 [15%]), and cytomegalovirus (34 [12%]; table 3). Among the 165 children with an infectious cause of death who were hospitalised for less than 48 h before death, the most common pathogens identified were *S pneumoniae* (45 [27%]), *K pneumoniae* (28 [17%]), and *Plasmodium falciparum* (25 [15%]; data not shown). Pathogens were most commonly associated with sepsis and lower respiratory tract infections (at any position in the causal chain; table 3), which were collectively associated with 73% of child deaths. The detailed cause-of-death information available for an infant with multiple contributing causes of death, including a congenital condition and at least one pathogen contributing to death, is shown in the appendix (p 19).

## Discussion

Pathology-based surveillance for child mortality in regions with the highest child mortality is beginning to identify the causes of stillbirths and neonatal and childhood deaths with unprecedented detail. We implemented real-time child mortality surveillance systems that identified and notified us of deaths among stillbirths and children aged younger than 5 years across five CHAMPS network sites in sub-Saharan Africa and Bangladesh. Around three-quarters of families who were approached consented to the MITS procedure, showing that MITS is feasible and can be readily accepted in high-mortality settings where complete diagnostic autopsies might not be feasible. These findings represent an early assessment and will evolve over time.

These data provide detailed causes of child mortality and stillbirths, yielding insights for the development of tools and interventions to reduce deaths in areas with high under-5 mortality rates. We observed that at least one pathogen contributed to 17% of stillbirths in this analysis. Similarly, lower respiratory tract infection was identified in the causal chain of 57% of deaths in children aged 1–59 months. By contrast, if only the underlying cause was considered, lower respiratory tract infection would have accounted for only 12% of child deaths. Preterm birth complications were common among neonatal deaths; however, infectious diseases contributed to mortality in 62% of those preterm births, providing multiple possible targets for life-saving interventions. Current burden of disease estimates would likely underestimate multiple contributors, many of which could be important targets for intervention. Such findings offer a new perspective on strategic child mortality prevention by highlighting pathways, such as lower respiratory tract infection and sepsis, which are common across many underlying cause categories and are potential targets for prevention.

The use of histopathology and immunohistochemistry in CHAMPS provides the capacity to distinguish infection with an inflammatory response from post-mortem artifacts. Post-mortem contamination by gastrointestinal and other normal flora is a key concern when interpreting MITS data. The eligibility criteria for MITS required that we were notified of deaths within 36 h (or ≤72 h if refrigerated) to limit the amount of tissue autolysis and potential post-mortem overgrowth.^30^ Additionally, the DeCoDe panels considered all data available for each case to define causes of death and did not include detected pathogens in the causal chain if other evidence did not provide conclusive support. For example, *K pneumoniae* was identified in the causal chain, associated with pneumonia, sepsis, and meningitis for a substantial number of deaths; however simply detecting *K pneumoniae* did not translate to determining that it caused the death. One of the key early findings from CHAMPS might be the importance of nosocomial and community-acquired *K pneumoniae*-associated morality. Although some studies have suggested the importance of *K pneumoniae* in child mortality,^31^, ^32^ this pathogen has not previously been considered to be a key contributory causal pathogen for child mortality; if consistent over time, CHAMPS data are likely to drive the development of new tools and strategies to target this pathogen to prevent child mortality.

Because of the potential for inherent variability for interpretation of data and diagnoses assigned by DeCoDe panels, we developed and applied standardised diagnostic criteria,^27^ coordinated standard operating procedures across sites and reviewed 10% of cases from each site by all of the panels, and adhered to ICD-10 and ICD-PM standards in assigning underlying cause and causal chain.

Cause of death determination in CHAMPS has some important limitations. Most of the initial cases included in this analysis of data obtained during the first 2 years of CHAMPS occurred in hospitals and health centres; thus, the findings presented in this study might not fully reflect the underlying populations from which cases arose. More than half of cases in this study originated from a single large tertiary hospital in Soweto, where the neonatal unit has had ongoing nosocomial gram-negative bacterial transmission (predominately *Acinetobacter*),^33^ and distribution of causes of death and care available for this population are likely to differ from that at the other sites. In April, 2018, the catchment area for CHAMPS in Soweto was reduced from the entire township to a nested HDSS population similar in size to other CHAMPS sites. With the accumulation of cases from other sites, from cases hospitalised less than 48 h thereby representing community acquired infections and increasingly from community settings in the future, the distribution of total cases across CHAMPS sites will become more geographically balanced and reflective of areas with high under-5 mortality in sub-Saharan Africa and south Asia. Cause-specific mortality fractions and pathogen prevalence derived from CHAMPS data should not yet be interpreted as statistically valid estimates beyond these sites.

Post-mortem studies can provide valuable data that were not otherwise available at the time of death, time of physician certification, or verbal autopsy. However, current CHAMPS testing and interview procedures do not systematically evaluate some potentially important causes, such as congenital heart disease, intracranial bleeding, some types of poisonings or toxic exposures, certain inborn errors of metabolism, genetic disorders without characteristic physical features, or sociocultural factors that impact child mortality; antemortem diagnosis of some of these conditions would differ depending on site resources. CHAMPS is investigating several methods to improve diagnostic accuracy for such causes of death. For example, CHAMPS diagnosis of prematurity was often based on documented birthweight when data on last maternal menstrual period were unavailable, which could misclassify babies who were small for gestational age; thus, assessment of methods to better estimate gestational age (including biomarkers) is planned. Other diagnostic methods under consideration include improved detection of maternal pre-eclampsia or eclampsia in the fetus or placenta and systematic screening for common environmental toxins and poisoning. For cases with localised lesions, the sensitivity of MITS for detecting infectious or other conditions might be less than 100%;^14^ however, the practice of collecting multiple specimens from each target organ decreases the likelihood that a diffuse process leading to death would be missed.

Post-mortem tissue sampling was accepted by many families in a wide variety of settings and resulted in high-resolution cause of death data. The use of MITS, combined with other data, and comprehensive assessment by expert panels provided greater detail on causes of death than traditional data sources such as verbal autopsy and vital registration. Over time, epidemiological and clinical insights are anticipated that would be unattainable with currently used methods. A single underlying cause of death is not sufficient to characterise precipitating, potentially preventable factors associated with mortality in settings where child mortality is high. The surveillance described in this study used a combination of novel and highly precise approaches, which provides the potential for new evidence that can be used to reduce preventable childhood mortality.

## Data sharing

CHAMPS data are available online.
