# Supplementary material for: Initial findings from a novel population-based child mortality surveillance approach: a descriptive study
Source: Lancet Glob Health. 2020 Jun 17;8(7):e909–19. doi: 10.1016/S2214-109X(20)30205-9 (PMC7303945; doi:10.1016/S2214-109X(20)30205-9)

THE LANCET
Global Health

Supplementary appendix

This appendix formed part of the original submission and has been peer reviewed. We post it as supplied by the authors.

Supplement to: Taylor AW, Blau DM, Bassat Q, et al. Initial findings from a novel population-based child mortality surveillance approach: a descriptive study. *Lancet Glob Health* 2020; **8:** e909–19.

APPENDIX: Initial findings from a novel population-based child mortality surveillance approach (CHAMPS): a descriptive study

Contents

Supplementary Table S1 CHAMPS Cause of Death Categories 2

Supplemental Table S2 Selected characteristics of CHAMPS-eligible, MITS-consented and non-MITS consented populations, by case type, CHAMPS Network (sites in Manhiça, Mozambique; Soweto, South Africa; Kisumu, Kenya; Bamako, Mali; and Baliakandi, Bangladesh), December 2016–December 2018 15

Supplemental Table S3. Number of listed causes of death, by selected case characteristics, CHAMPS Network (sites in Manhiça, Mozambique; Soweto, South Africa; Kisumu, Kenya; Bamako, Mali; and Baliakandi, Bangladesh), December 2016–December 2018 17

Supplementary Table S4: ICD-PM maternal condition group Main maternal conditions included in group* 18

Supplementary Figure S1: Illustrative cases 19

Figure S2. 20

Figure S3 20

Figure S4 (annotated) 21

Figure S4. Case 3 (not annotated) 22

Supplementary Table S1 CHAMPS Cause of Death Categories

Anemias

- D64 Other anaemias
- D64.9 Anaemia, unspecified

BIrth trauma

- P15.8 Other specified birth injuries
- P15.9 Birth injury, unspecified

Cancer

- C22.2 Malignant neoplasm: Hepatoblastoma
- C49.9 Malignant neoplasm: Connective and soft tissue, unspecified
- C74.9 Malignant neoplasm: Adrenal gland, unspecified

Cesarean delivery

- P03.4 Fetus and newborn affected by caesarean delivery

Chorioamnionitis and membrane complications

- P02.7 Fetus and newborn affected by chorioamnionitis

Congenital birth defects

- G71.2 Congenital myopathies
- G80.9 Cerebral palsy, unspecified
- Q00.0 Anencephaly
- Q02 Microcephaly
- Q03 Congenital hydrocephalus
- Q03.1 Atresia of foramina of Magendie and Luschka
- Q03.9 Congenital hydrocephalus, unspecified
- Q04.2 Holoprosencephaly
- Q04.6 Congenital cerebral cysts
- Q05 Spina bifida
- Q05.9 Spina bifida, unspecified
- Q11.1 Other anophthalmos
- Q21.0 Ventricular septal defect
- Q21.1 Atrial septal defect

Q21.3 Tetralogy of Fallot
Q23.4 Hypoplastic left heart syndrome
Q24.8 Other specified congenital malformations of heart
Q24.9 Congenital malformation of heart, unspecified
Q25.1 Coarctation of aorta
Q31.0 Web of larynx
Q31.1 Congenital subglottic stenosis
Q35 Cleft palate
Q35.9 Cleft palate, unspecified
Q37.8 Unspecified cleft palate with bilateral cleft lip
Q39.1 Atresia of oesophagus with tracheo-oesophageal fistula
Q41.1 Congenital absence, atresia and stenosis of jejunum
Q42.0 Congenital absence, atresia and stenosis of rectum with fistula
Q43.3 Congenital malformations of intestinal fixation
Q44.2 Atresia of bile ducts
Q44.7 Other congenital malformations of liver
Q61.3 Polycystic kidney, unspecified
Q62.0 Congenital hydronephrosis
Q64.2 Congenital posterior urethral valves
Q64.9 Congenital malformation of urinary system, unspecified
Q75.3 Macrocephaly
Q77.1 Thanatophoric short stature
Q78.9 Osteochondrodysplasia, unspecified
Q79.0 Congenital diaphragmatic hernia
Q79.2 Exomphalos
Q79.3 Gastroschisis
Q81.9 Epidermolysis bullosa, unspecified
Q87 Other specified congenital malformation syndromes affecting multiple systems
Q87.2 Congenital malformation syndromes predominantly involving limbs
Q87.8 Other specified congenital malformation syndromes, not elsewhere classified
Q89 Other congenital malformations, not elsewhere classified
Q89.7 Multiple congenital malformations, not elsewhere classified
Q89.9 Congenital malformation, unspecified

Q90.9 Down syndrome, unspecified
Q91.3 Edwards syndrome, unspecified
Q91.7 Patau syndrome, unspecified
Q93.2 Chromosome replaced with ring or dicentric
Q98.0 Klinefelter syndrome karyotype 47, XXY
Q99.9 Chromosomal abnormality, unspecified

Congenital infection

P23.2 Congenital pneumonia due to staphylococcus
P23.3 Congenital pneumonia due to streptococcus, group B
P23.4 Congenital pneumonia due to Escherichia coli
P23.6 Congenital pneumonia due to other bacterial agents
P23.8 Congenital pneumonia due to other organisms
P23.9 Congenital pneumonia, unspecified
P35.1 Congenital cytomegalovirus infection
P37.2 Neonatal (disseminated) listeriosis
P37.5 Neonatal candidiasis
P38 Omphalitis of newborn with or without mild haemorrhage
P39.9 Infection specific to the perinatal period, unspecified

Diarrheal Diseases

A03 Shigellosis
A04.4 Other intestinal Escherichia coli infections
A04.5 Campylobacter enteritis
A04.8 Other specified bacterial intestinal infections
A07.1 Giardiasis [lambliasis]
A08.0 Rotaviral enteritis
A08.1 Acute gastroenteropathy due to Norwalk agent
A08.2 Adenoviral enteritis
A08.3 Other viral enteritis
A08.5 Other specified intestinal infections
A09 Other gastroenteritis and colitis of infectious and unspecified origin
A09.0 Other and unspecified gastroenteritis and colitis of infectious origin
A09.9 Gastroenteritis and colitis of unspecified origin
K52.9 Noninfective gastroenteritis and colitis, unspecified

R11 Nausea and vomiting

Epilepsy

G41.9 Status epilepticus, unspecified

Heart Diseases

I33.0 Acute and subacute infective endocarditis
I42.0 Dilated cardiomyopathy
I42.9 Cardiomyopathy, unspecified
I50 Heart failure
I51.9 Heart disease, unspecified

HIV

B20 Human immunodeficiency virus [HIV] disease resulting in infectious and parasitic diseases
B20.1 HIV disease resulting in other bacterial infections
B20.3 HIV disease resulting in other viral infections
B20.7 HIV disease resulting in multiple infections
B20.8 HIV disease resulting in other infectious and parasitic diseases
B22 Human immunodeficiency virus [HIV] disease resulting in other specified diseases
B22.2 HIV disease resulting in wasting syndrome
B22.7 HIV disease resulting in multiple diseases classified elsewhere
B23 Human immunodeficiency virus [HIV] disease resulting in other conditions
B23.8 HIV disease resulting in other specified conditions

Injury

S06.5 Traumatic subdural haemorrhage
S07 Crushing injury of head
T20 Burn and corrosion of head and neck
T31.0 Burns involving less than 10% of body surface
T31.1 Burns involving 10-19% of body surface
T31.2 Burns involving 20-29% of body surface
T31.3 Burns involving 30-39% of body surface
T31.5 Burns involving 50-59% of body surface
T71 Asphyxiation
T74.1 Physical abuse
V03.9 Unspecified whether traffic or nontraffic accident

Kidney Disease

N17.9 Acute renal failure, unspecified

Liver disease

B18.2 Chronic viral hepatitis C
E88.8 Other specified metabolic disorders
K72.0 Acute and subacute hepatic failure
K72.1 Chronic hepatic failure
K72.9 Hepatic failure, unspecified
K74.5 Biliary cirrhosis, unspecified
K75.9 Inflammatory liver disease, unspecified
K76.5 Hepatic veno-occlusive disease
K83.1 Obstruction of bile duct

Lower respiratory infections

A37 Whooping cough
B20.6 HIV disease resulting in Pneumocystis jirovecii pneumonia
B22.1 HIV disease resulting in lymphoid interstitial pneumonitis
B25.0 Cytomegaloviral pneumonitis
J10.0 Influenza with pneumonia, seasonal influenza virus identified
J12.0 Adenoviral pneumonia
J12.1 Respiratory syncytial virus pneumonia
J12.2 Parainfluenza virus pneumonia
J12.8 Other viral pneumonia
J13 Pneumonia due to Streptococcus pneumoniae
J14 Pneumonia due to Haemophilus influenzae
J15.0 Pneumonia due to Klebsiella pneumoniae
J15.1 Pneumonia due to Pseudomonas
J15.2 Pneumonia due to staphylococcus
J15.4 Pneumonia due to other streptococci
J15.5 Pneumonia due to Escherichia coli
J15.6 Pneumonia due to other Gram-negative bacteria
J15.8 Other bacterial pneumonia
J15.9 Bacterial pneumonia, unspecified
J16.8 Pneumonia due to other specified infectious organisms
J17.1 Pneumonia in viral diseases classified elsewhere

J17.3 Pneumonia in parasitic diseases
J18 Pneumonia, organism unspecified
J18.0 Bronchopneumonia, unspecified
J18.9 Pneumonia, unspecified
J21.9 Acute bronchiolitis, unspecified
J69.0 Pneumonitis due to food and vomit
J85.1 Abscess of lung with pneumonia
J86.9 Pyothorax without fistula
W78 Inhalation of gastric contents

Malaria

B50 Plasmodium falciparum malaria
B50.0 Plasmodium falciparum malaria with cerebral complications
B50.8 Other severe and complicated Plasmodium falciparum malaria

Malnutrition

E40 Kwashiorkor
E41 Nutritional marasmus
E42 Marasmic kwashiorkor
E43 Unspecified severe protein-energy malnutrition
E44.0 Moderate protein-energy malnutrition
E46 Unspecified protein-energy malnutrition

Malpresentation before labor

P01.7 Fetus and newborn affected by malpresentation before labour

Maternal HIV

B24 Unspecified human immunodeficiency virus [HIV] disease
Z20.6 Contact with and exposure to human immunodeficiency virus [HIV]

Maternal hypertension

O14.2 HELLP syndrome

Maternal infection

P00.2 Fetus and newborn affected by maternal infectious and parasitic diseases

Measles

B05.2 Measles complicated by pneumonia
B05.8 Measles with other complications

Meningitis/Encephalitis

- A85.1 Adenoviral encephalitis
- A87.8 Other viral meningitis
- B37.5 Candidal meningitis
- G00.1 Pneumococcal meningitis
- G00.2 Streptococcal meningitis
- G00.3 Staphylococcal meningitis
- G00.8 Other bacterial meningitis
- G01 Meningitis in bacterial diseases classified elsewhere
- G03.9 Meningitis, unspecified
- G04.8 Other encephalitis, myelitis and encephalomyelitis

Motor neuron disease

- G12.9 Spinal muscular atrophy, unspecified

Neonatal aspiration syndromes

- P24.0 Neonatal aspiration of meconium
- P24.9 Neonatal aspiration syndrome, unspecified

Neonatal encephalopathy

- P91 Other disturbances of cerebral status of newborn
- P91.0 Neonatal cerebral ischaemia
- P91.2 Neonatal cerebral leukomalacia
- P91.8 Other specified disturbances of cerebral status of newborn

Neonatal preterm birth complications

- P07.0 Extremely low birth weight
- P07.1 Other low birth weight
- P07.18 Neonatal preterm birth complications
- P07.3 Other preterm infants
- P22.0 Respiratory distress syndrome of newborn
- P22.9 Respiratory distress of newborn, unspecified
- P25.1 Pneumothorax originating in the perinatal period
- P25.2 Pneumomediastinum originating in the perinatal period
- P26 Pulmonary haemorrhage originating in the perinatal period
- P26.8 Other pulmonary haemorrhages originating in the perinatal period
- P26.9 Unspecified pulmonary haemorrhage originating in the perinatal period
- P27.1 Bronchopulmonary dysplasia originating in the perinatal period

P28.0 Primary atelectasis of newborn
P77 Necrotizing enterocolitis of fetus and newborn

Neonatal sepsis

P36.0 Sepsis of newborn due to streptococcus, group B
P36.1 Sepsis of newborn due to other and unspecified streptococci
P36.2 Sepsis of newborn due to Staphylococcus aureus
P36.3 Sepsis of newborn due to other and unspecified staphylococci
P36.4 Sepsis of newborn due to Escherichia coli
P36.5 Sepsis of newborn due to anaerobes
P36.8 Other bacterial sepsis of newborn
P36.9 Bacterial sepsis of newborn, unspecified

Obstructed labor and fetal malpresentation

P03.1 Fetus and newborn affected by other malpresentation, malposition and disproportion during labour and delivery

Other

F89 Unspecified disorder of psychological development
K92.0 Haematemesis
K92.9 Disease of digestive system, unspecified
R19.8 Other specified symptoms and signs involving the digestive system and abdomen
R62.8 Other lack of expected normal physiological development
T80.2 Infections following infusion, transfusion and therapeutic injection
T81 Complications of procedures, not elsewhere classified
T81.4 Infection following a procedure, not elsewhere classified
T85.7 Infection and inflammatory reaction due to other internal prosthetic devices, implants and grafts
T88.3 Malignant hyperthermia due to anaesthesia
T88.9 Complication of surgical and medical care, unspecified
Y63.8 Failure in dosage during other surgical and medical care
Y84.0 Cardiac catheterization

Other disorders of fluid, electrolyte and acid-base balance

E87.0 Hyperosmolality and hypernatraemia
R57.1 Hypovolaemic shock

Other endocrine, metabolic, blood, and immune disorders

D65 Disseminated intravascular coagulation [defibrination syndrome]
D66 Hereditary factor VIII deficiency

D69.9 Haemorrhagic condition, unspecified
E16.2 Hypoglycaemia, unspecified
E22.2 Syndrome of inappropriate secretion of antidiuretic hormone

Other gastrointestinal disease

K55.0 Acute vascular disorders of intestine

Other immunodeficiencies

D82.1 Di George syndrome
D84.9 Immunodeficiency, unspecified

Other infections

A39.2 Acute meningococcaemia
B00.7 Disseminated herpesviral disease
B25.8 Other cytomegaloviral diseases
B25.9 Cytomegaloviral disease, unspecified
B33.8 Other specified viral diseases
B34.0 Adenovirus infection, unspecified site
B37.1 Pulmonary candidiasis
B37.8 Candidiasis of other sites
B44.7 Disseminated aspergillosis
B59 Pneumocystosis
G06.0 Intracranial abscess and granuloma
H66.3 Other chronic suppurative otitis media
H66.4 Suppurative otitis media, unspecified
I51.4 Myocarditis, unspecified
K65.0 Acute peritonitis
L03 Cellulitis
M00 Pyogenic arthritis
M86.9 Osteomyelitis, unspecified

Other injury

T07 Unspecified multiple injuries
T17.9 Foreign body in respiratory tract, part unspecified

Other labor and delivery complications

O69.9 Labour and delivery complicated by cord complication, unspecified

Other neonatal disorders

P05.0 Light for gestational age
P05.9 Slow fetal growth, unspecified
P08.0 Exceptionally large baby
P08.1 Other heavy for gestational age infants
P29.0 Neonatal cardiac failure
P29.2 Neonatal hypertension
P29.3 Persistent fetal circulation
P51.0 Massive umbilical haemorrhage of newborn
P52.0 Intraventricular (nontraumatic) haemorrhage, grade 1, of fetus and newborn
P52.1 Intraventricular (nontraumatic) haemorrhage, grade 2, of fetus and newborn
P52.2 Intraventricular (nontraumatic) haemorrhage, grade 3, and grade 4 of fetus and newborn
P52.3 Unspecified intraventricular (nontraumatic) haemorrhage of fetus and newborn
P52.4 Intracerebral (nontraumatic) haemorrhage of fetus and newborn
P52.8 Other intracranial (nontraumatic) haemorrhages of fetus and newborn
P52.9 Intracranial (nontraumatic) haemorrhage of fetus and newborn, unspecified
P55.1 ABO isoimmunization of fetus and newborn
P55.9 Haemolytic disease of fetus and newborn, unspecified
P57.0 Kernicterus due to isoimmunization
P57.9 Kernicterus, unspecified
P59.9 Neonatal jaundice, unspecified
P60 Disseminated intravascular coagulation of fetus and newborn
P61.0 Transient neonatal thrombocytopenia
P70.4 Other neonatal hypoglycaemia
P78.0 Perinatal intestinal perforation
P80.9 Hypothermia of newborn, unspecified
P83.2 Hydrops fetalis not due to haemolytic disease
P90 Convulsions of newborn

Other neurological disorders

G40.9 Epilepsy, unspecified
G91.0 Communicating hydrocephalus
G91.8 Other hydrocephalus
G91.9 Hydrocephalus, unspecified
G93.5 Compression of brain

G96.9 Disorder of central nervous system, unspecified
R56.8 Other and unspecified convulsions
S06.2 Diffuse brain injury

Other respiratory disease

I27.2 Other secondary pulmonary hypertension
J35.3 Hypertrophy of tonsils with hypertrophy of adenoids
J44.8 Other specified chronic obstructive pulmonary disease
J46 Status asthmaticus
J47 Bronchiectasis
J69.8 Pneumonitis due to other solids and liquids
J84.9 Interstitial pulmonary disease, unspecified
J93 Pneumothorax
J93.9 Pneumothorax, unspecified
J98.4 Other disorders of lung
R04.8 Haemorrhage from other sites in respiratory passages
R06.8 Other and unspecified abnormalities of breathing
Z87.0 Personal history of diseases of the respiratory system

Other skin and subcutaneous diseases

L02.0 Cutaneous abscess, furuncle and carbuncle of face
L26 Exfoliative dermatitis
L51.2 Toxic epidermal necrolysis [Lyell]
L98.4 Chronic ulcer of skin, not elsewhere classified

Paralytic ileus and intestinal obstruction

K56.1 Intussusception
K56.2 Volvulus
K56.6 Other and unspecified intestinal obstruction
K56.7 Ileus, unspecified

Perinatal asphyxia/hypoxia

P20 Intrauterine hypoxia
P20.0 Intrauterine hypoxia first noted before onset of labour
P20.1 Intrauterine hypoxia first noted during labour and delivery
P20.9 Intrauterine hypoxia, unspecified
P21.0 Severe birth asphyxia

P21.1 Mild and moderate birth asphyxia
P21.9 Birth asphyxia, unspecified

Placental complications

P02.2 Fetus and newborn affected by other and unspecified morphological and functional abnormalities of placenta
P02.3 Fetus and newborn affected by placental transfusion syndromes

Poisoning

T41.2 Poisoning: Other and unspecified general anaesthetics
T45.1 Poisoning: Antineoplastic and immunosuppressive drugs
T47.4 Poisoning: Other laxatives
T55 Toxic effect of soaps and detergents
T60.0 Toxic effect: Organophosphate and carbamate insecticides

Premature Rupture of membranes

P01.1 Fetus and newborn affected by premature rupture of membranes

Road injuries

V09.3 Pedestrian injured in unspecified traffic accident

Sepsis

A02.1 Salmonella sepsis
A40 Streptococcal sepsis
A40.0 Sepsis due to streptococcus, group A
A40.3 Sepsis due to Streptococcus pneumoniae
A41.0 Sepsis due to Staphylococcus aureus
A41.3 Sepsis due to Haemophilus influenzae
A41.4 Sepsis due to anaerobes
A41.5 Sepsis due to other Gram-negative organisms
A41.8 Other specified sepsis
A41.9 Sepsis, unspecified
A48.3 Toxic shock syndrome
B37.7 Candidal sepsis
G93.4 Encephalopathy, unspecified

Sickle cell disorders

D57.0 Sickle-cell anaemia with crisis
D57.1 Sickle-cell anaemia without crisis

Spontaneous abortion

O03 Spontaneous abortion

Sudden infant death syndrome

R95 Sudden infant death syndrome

Syphilis

A50 Congenital syphilis

A50.9 Congenital syphilis, unspecified

Tuberculosis

A17.0 Tuberculous meningitis

A18.8 Tuberculosis of other specified organs

A19.1 Acute miliary tuberculosis of multiple sites

A19.9 Miliary tuberculosis, unspecified

Umbilical cord complications

P02.4 Fetus and newborn affected by prolapsed cord

P02.5 Fetus and newborn affected by other compression of umbilical cord

Undetermined

P95 Fetal death of unspecified cause

R96 Other sudden death, cause unknown

R99 Other ill-defined and unspecified causes of mortality

Upper respiratory infections

J05.0 Acute obstructive laryngitis [croup]

J06.9 Acute upper respiratory infection, unspecified

Supplemental Table S2 Selected characteristics of CHAMPS-eligible, MITS-consented and non-MITS consented populations, by case type, CHAMPS Network (sites in Manhiça, Mozambique; Soweto, South Africa; Kisumu, Kenya; Bamako, Mali; and Baliakandi, Bangladesh), December 2016–December 2018

	MITS case (N=933)	Non-MITS case (N=1179)	Not enrolled (N=273)	Total (N=2385)
**CHAMPS Site**				
South Africa	464 (50%)	106 (9%)	188 (69%)	758 (32%)
Mozambique	146 (16%)	285 (24%)	4 (1%)	435 (18%)
Kenya	182 (20%)	126 (11%)	14 (5%)	322 (14%)
Mali	95 (10%)	347 (29%)	1 (0%)	443 (19%)
Bangladesh	46 (5%)	315 (27%)	66 (24%)	427 (18%)
**Age group**				
Stillbirth	180 (19%)	411 (35%)	65 (24%)	656 (28%)
Death in the first 24 hours	162 (17%)	150 (13%)	42 (15%)	354 (15%)
Early Neonate (1 to 6 days)	185 (20%)	148 (13%)	76 (28%)	409 (17%)
Late Neonate (7 to 27 days)	102 (11%)	87 (7%)	28 (10%)	217 (9%)
Infant (28 days to less than 12 months)	174 (19%)	204 (17%)	39 (14%)	417 (17%)
Child (12 months to less than 60 Months)	130 (14%)	179 (15%)	23 (8%)	332 (14%)
Missing	0 (0%)	0 (0%)	0 (0%)	0 (0%)
**Religion of primary caretaker**				
N-Miss	0	11	0	11
Muslim	135 (14%)	623 (53%)	65 (24%)	823 (35%)
Christian	557 (60%)	394 (34%)	133 (49%)	1084 (46%)
African traditional religion	45 (5%)	52 (4%)	0 (0%)	97 (4%)
Other or no religion	196 (21%)	99 (8%)	75 (27%)	370 (16%)
**Sex of deceased**				
Female	407 (44%)	507 (43%)	109 (40%)	1023 (43%)
Indeterminate	2 (0%)	0 (0%)	0 (0%)	2 (0%)
Male	524 (56%)	660 (56%)	164 (60%)	1348 (57%)
Unknown	0 (0%)	12 (1%)	0 (0%)	12 (1%)
**Hours from death to notification**				
<=24 hr	813 (87%)	565 (48%)	227 (83%)	1605 (67%)
>24 hr	120 (13%)	614 (52%)	46 (17%)	780 (33%)
Missing	0 (0%)	0 (0%)	0 (0%)	0 (0%)
**Hours from death to MITS procedure**				
<=24 hours	616 (66%)	0 (0%)	0 (0%)	616 (26%)
>24 hours	316 (34%)	0 (0%)	0 (0%)	316 (13%)
Missing or Not Applicable	1 (0%)	1179 (100%)	273 (100%)	1453 (61%)
**Location and timing of death**				
Facility, <=24 hours after admission	268 (29%)	106 (9%)	0 (0%)	374 (16%)
Facility, >24 hours after admission	572 (61%)	646 (55%)	251 (92%)	1469 (62%)
Community	93 (10%)	427 (36%)	22 (8%)	542 (23%)

Supplemental Table S3. Number of listed causes of death, by selected case characteristics, CHAMPS Network (sites in Manhiça, Mozambique; Soweto, South Africa; Kisumu, Kenya; Bamako, Mali; and Baliakandi, Bangladesh), December 2016–December 2018

	0 (N=21)	1 (N=327)	2 (N=219)	3 (N=174)	4 (N=111)	5 (N=57)	6 (N=14)	7 (N=10)	Total (N=933)
**Age group**									
1–59 months	6 (2%)	68 (22%)	82 (27%)	69 (23%)	44 (14%)	23 (8%)	7 (2%)	5 (2%)	304 (100%)
Neonate	7 (2%)	112 (25%)	116 (26%)	102 (23%)	66 (15%)	34 (8%)	7 (2%)	5 (1%)	449 (100%)
Stillbirth	8 (4%)	147 (82%)	21 (12%)	3 (2%)	1 (1%)	0 (0%)	0 (0%)	0 (0%)	180 (100%)
**CHAMPS Site**									
Bangladesh	1 (2%)	19 (41%)	12 (26%)	10 (22%)	4 (9%)	0 (0%)	0 (0%)	0 (0%)	46 (100%)
Kenya	7 (4%)	96 (53%)	41 (23%)	23 (13%)	13 (7%)	2 (1%)	0 (0%)	0 (0%)	182 (100%)
Mali	6 (6%)	46 (48%)	18 (19%)	15 (16%)	6 (6%)	2 (2%)	0 (0%)	2 (2%)	95 (100%)
Mozambique	5 (3%)	89 (61%)	32 (22%)	12 (8%)	8 (5%)	0 (0%)	0 (0%)	0 (0%)	146 (100%)
South Africa	2 (0%)	77 (17%)	116 (25%)	114 (25%)	80 (17%)	53 (11%)	14 (3%)	8 (2%)	464 (100%)
**Location and timing of death**									
Community	5 (5%)	43 (46%)	26 (28%)	15 (16%)	3 (3%)	0 (0%)	0 (0%)	1 (1%)	93 (100%)
Facility, ≤24 hours after admission	8 (3%)	104 (39%)	85 (32%)	41 (15%)	23 (9%)	6 (2%)	1 (0%)	0 (0%)	268 (100%)
Facility, >24 hours after admission	8 (1%)	180 (31%)	108 (19%)	118 (21%)	85 (15%)	51 (9%)	13 (2%)	9 (2%)	572 (100%)

Supplementary Table S4: ICD-PM maternal condition group Main maternal conditions included in group*

M1: Complications of placenta, cord and membranes

1. placenta praevia
2. other forms of placental separation and haemorrhage
3. placental dysfunction, infarction, insufficiency
4. fetal-placental transfusion syndromes
5. prolapsed cord, other compression of umbilical cord
6. chorioamnionitis
7. other complications of membranes

M2: Maternal complications of pregnancy

1. incompetent cervix
2. preterm rupture of membranes
3. oligohydramnios/polyhydramnios
4. ectopic pregnancy
5. multiple pregnancy
6. maternal death
7. malpresentation before labour
8. other complications of pregnancy

M3: Other complications of labour and delivery

1. breech delivery and extraction
2. other malpresentation, malposition and disproportion during labour and delivery
3. forceps delivery/vacuum extraction
4. caesarean delivery
5. precipitate delivery
6. preterm labour and delivery
7. other complications of labour and delivery, including termination of pregnancy

M4: Maternal medical and surgical conditions

1. pre-eclampsia, eclampsia
2. gestational hypertension
3. other hypertensive disorders
4. renal and urinary tract diseases
5. infectious and parasitic disease
6. circulatory and respiratory disease
7. nutritional disorders
8. injury
9. surgical procedure
10. other medical procedures
11. maternal diabetes, including gestational diabetes
12. maternal anaesthesia and analgesia
13. maternal medication
14. tobacco/alcohol/drugs of addiction
15. nutritional chemical substances
16. environmental chemical substances
17. unspecified maternal condition

M5: No maternal condition

1. no maternal condition identified (healthy mother)

Supplementary Figure S1: Illustrative cases

Case 1: Stillbirth

A 35-week estimated gestational age (EGA) stillborn female with a birth weight of 2.4 kg from a twin pregnancy. The mother was a 20-year-old woman who had been diagnosed with HIV prior to pregnancy and had attended 4 antenatal care visits but did not have a recorded syphilis test. Post-mortem HIV, malaria and TB testing on the deceased were negative. TAC of blood and CSF was strongly positive for *Treponema pallidum*. Immunohistochemistry demonstrated the spirochetes in liver, CNS and placenta. Study staff on site reported back the results to the mother of the stillbirth and was able to confirm the positive syphilis test and treat her and the remaining family according to national guidelines.

Case 2: Neonate

A female neonate with a low birth weight of 1.1 kg was admitted to the newborn nursery immediately after vaginal delivery for prematurity and respiratory distress syndrome. On admission, physical examination was normal with no congenital abnormalities detected. Treatment included oxygen via nasal catheter, dextrose, Vitamin K, crystalline penicillin and gentamicin. The day prior to death the neonate developed jaundice and fever. On postnatal day eight, the fever continued with diffuse coarse crepitations bilaterally, and the patient expired. Blood and CSF collected postmortem were positive by culture and TAC for *Klebsiella pneumoniae*. Lung, liver and CNS tissue were also positive for *K. pneumoniae* by immunohistochemistry. The DeCoDe panel assigned *Klebsiella* sepsis as the immediate cause of death and low birth weight as the underlying cause (prematurity was routinely coded as low birth weight). A verbal autopsy was not available on this neonate.

Case 3: 1–59 months old

A 17-month-old presented to a local hospital with a 4-day history of vomiting and subjective fever. On admission the child was noted to be anemic and in respiratory distress and shock. Despite aggressive fluid and oxygen treatment, the child desaturated and could not be resuscitated. *Streptococcus pneumoniae* was positive by culture and TAC in blood and CSF and in CNS tissue by immunohistochemistry. Lung TAC was also positive for *S pneumoniae*, *Klebsiella pneumoniae* and *Haemophilus influenzae* (non-B); immunohistochemistry was only positive for *S. pneumoniae*. Sickled erythrocytes were noted histologically in the liver, previous diagnosis of sickle cell disease was not known. The immediate cause of death determined for this case was pneumococcal sepsis with sickle cell disease as the underlying cause.

Figure S2.

Stillbirth due to syphilis. Panel shows abundant spirochetes within the liver tissue highlighted by T. pallidum immunohistochemistry (immunohistochemical assay with Treponema pallidum polyclonal antibody)

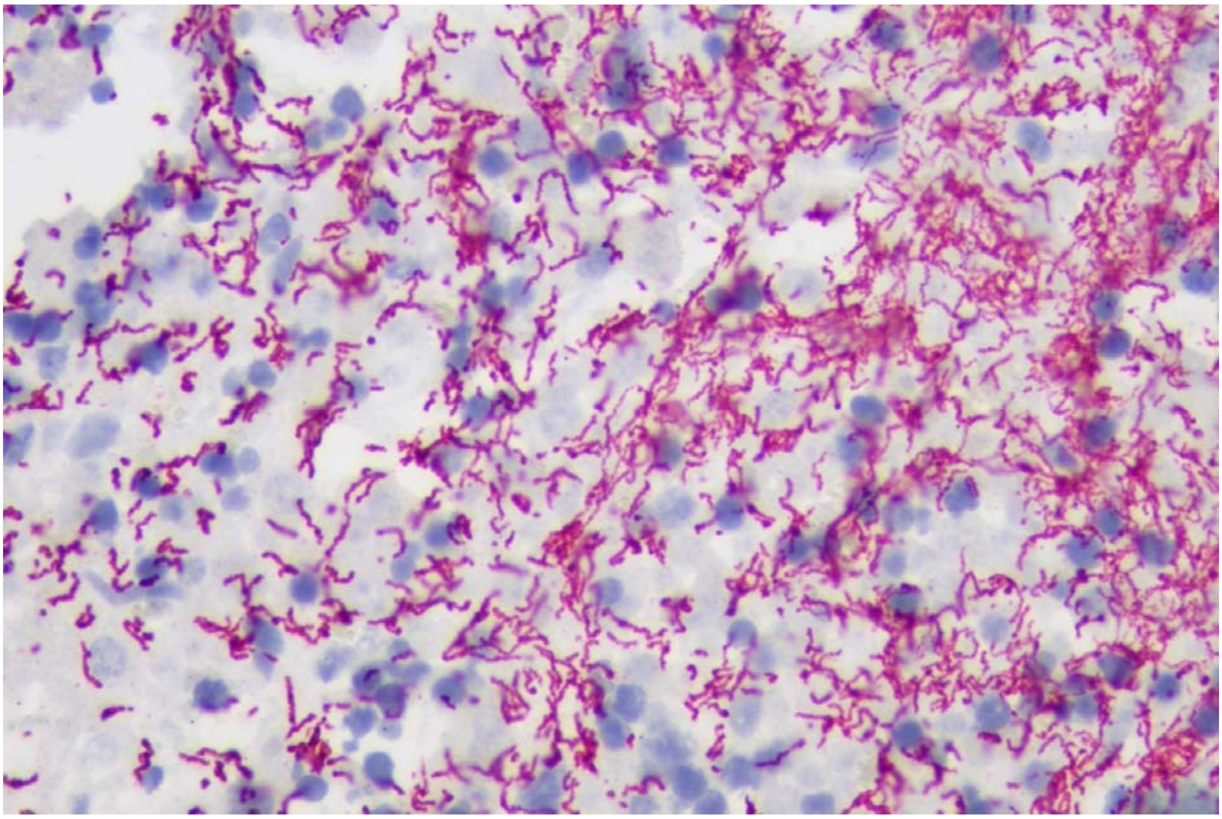

Figure S3- Neutrophilic meningitis caused by *Klebsiella pneumoniae*: Panel shows high magnification of abundant bacterial antigens in the meninges by immunohistochemistry (immunohistochemical assay with *Klebsiella pneumoniae* polyclonal antibody).

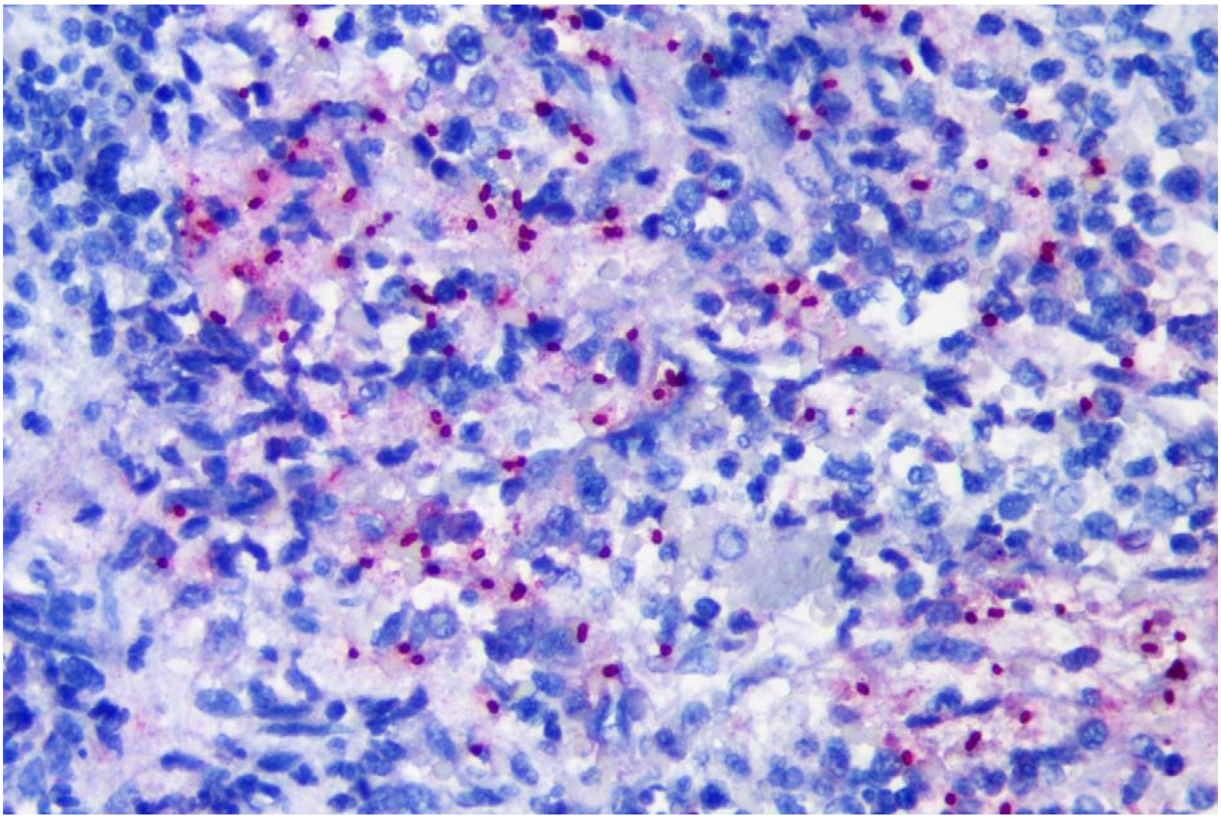

Figure S4 (annotated)- Pneumococcal sepsis with sickle cell disease: Panel shows microscopic examination of the liver shows sickle cell aggregates in sinusoidal spaces; hematoxylin-eosin stain.

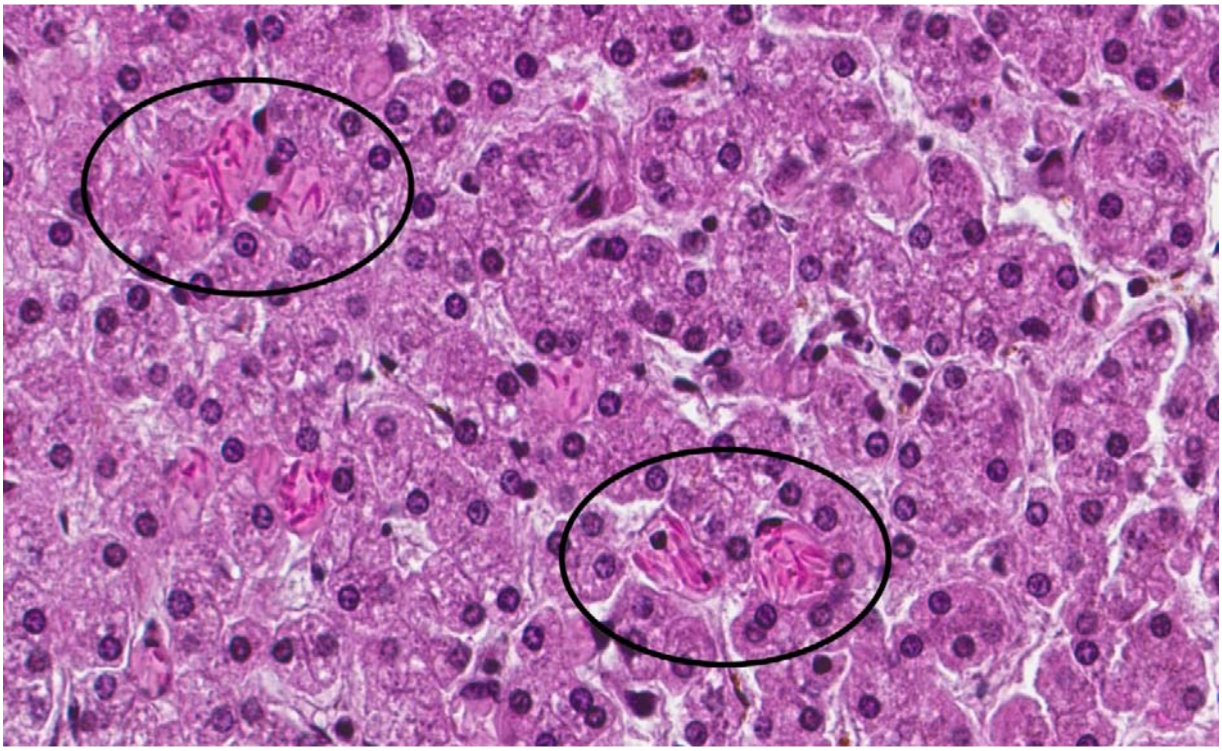

Figure S4. Case 3 (not annotated)

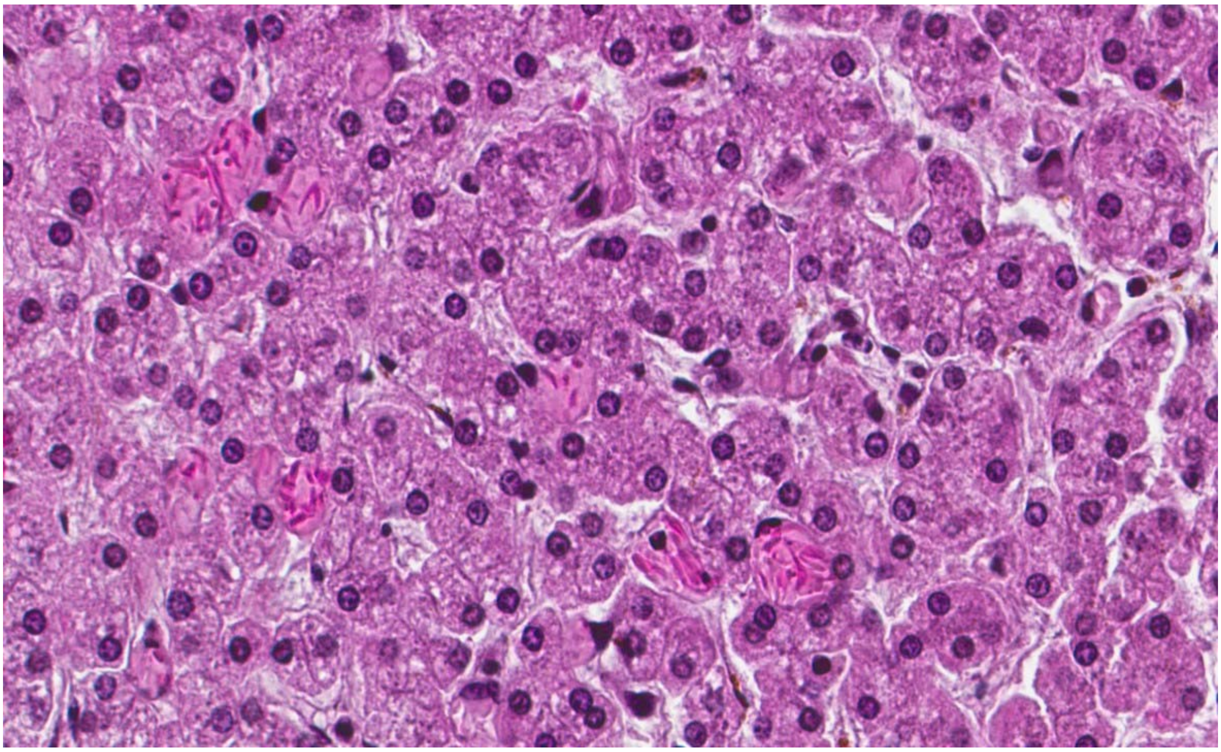

Supplement: Supplementary appendix [file mmc1.pdf]
